# Design, synthesis, molecular modelling, and biological evaluation of novel substituted pyrimidine derivatives as potential anticancer agents for hepatocellular carcinoma

**DOI:** 10.1080/14756366.2019.1612889

**Published:** 2019-05-23

**Authors:** Naglaa Mohamed Ahmed, Mahmoud Youns, Moustafa Khames Soltan, Ahmed Mohammed Said

**Affiliations:** a Pharmaceutical Organic Chemistry Department, Faculty of Pharmacy, Helwan University, Cairo, Egypt;; b Biochemistry Department, Faculty of Pharmacy, Helwan University, Cairo, Egypt;; c Department of Functional Genome Analysis, German Cancer Research Center (DKFZ), Heidelberg, Germany;; d Medicinal Chemistry Department, Faculty of Pharmacy, Zagazig University, Zagazig, Egypt;; e Oman College of Health Sciences, Muscat, Sultanate of Oman;; f Department of Chemistry, University at Buffalo, The State University of New York, Buffalo, NY, USA

**Keywords:** Pyrimidine, pyrazoline, anthracene, liver cancer, apoptosis, caspase 3/7

## Abstract

New anticancer agents are highly needed to overcome cancer cell resistance. A novel series of pyrimidine pyrazoline-anthracene derivatives (PPADs) (**4a-t)** were designed and synthesised. The anti-liver cancer activity of all compounds was screened *in vitro* against two hepatocellular carcinoma (HCC) cell lines (HepG2 and Huh-7) as well as normal fibroblast cells by resazurin assay. The designed compounds **4a-t** showed a broad-spectrum anticancer activity against the two cell lines and their activity was more prominent on cancer compared to normal cells. Compound **4e** showed high potency against HepG2 and Huh-7 cell lines (**(**IC_50_=5.34 and 6.13 μg/mL, respectively) comparable to that of doxorubicin (DOX) activities. A structure activity relationship (SAR) has been investigated and compounds **4e**, **4i**, **4m,** and **4q** were the most promising anticancer agents against tested cell lines. These compounds induced apoptosis in HepG2 and Huh-7 cells through significant activation of caspase 3/7 at all tested concentrations. In conclusion, **4e** could be a potent anticancer drug.

## Graphical Abstract

**Figure:**
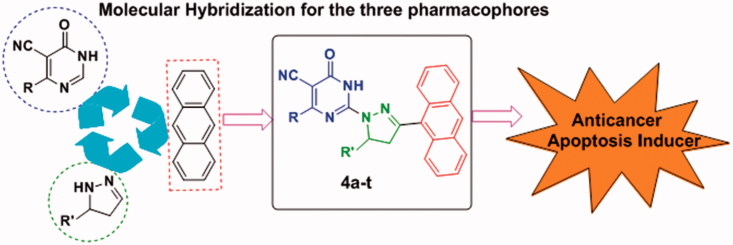

## Introduction 

Cancer (called tumour) is the result of uncontrolled growth and spread of abnormal cells^1^. Cancer is considered the leading cause of death worldwide based on the world health organisation (WHO) statistics. The most common types of cancer are lung, stomach, liver, colorectal, and female breast cancers. Hepatocellular carcinoma (HCC), one of the most common cancer types, is a primary malignancy of the liver and occurs predominantly in patients with underlying chronic liver disease. The mortality rate of HCC has significantly increased worldwide. In 2018, the American Cancer Society estimated that new liver cancer patients in USA to be over 40,000 cases^2^. One of the most common risk factors for developing HCC is the chronic viral infection with hepatitis C virus (HCV) or hepatitis B virus (HBV) or both^3^. There are many therapeutic strategies for targeting HCC. This includes chemotherapy, radiotherapy, and immunotherapy. These types of treatment are often accompanied by high systemic toxicity and sometimes drug resistance. Therefore, new therapeutic agents are highly needed to overcome this tumour drug resistance^4^.

**Figure 1.**
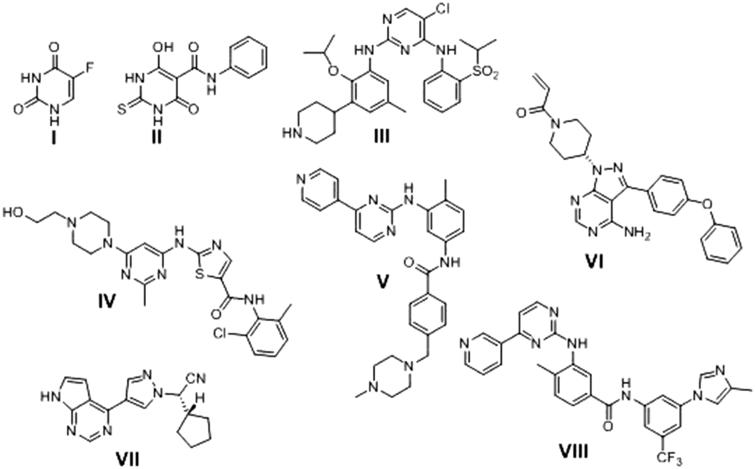
Anticancer drugs have pyrimidine ring in their structure.

Pyrimidine ring is one of the mostly used heterocyclic scaffolds in medicinal chemistry. Pyrimidine derivatives have been well recognised for their therapeutic applications i.e. antiviral^5^ ^,^ ^6^, antibacterial^7^ ^,^ ^8^, antifungal^9^, anti-inflammatory^10^, COX-2 inhibitors^11^, antioxidant^12^, antithyroid^13^, anticonvulsant^14^, and anti-diabetic^15^. Pyrimidine ring is also an integral part of DNA nucleic acid composition. Various drugs containing pyrimidine nucleus are being used as potent anticancer agents through different mechanisms of action i.e. 5-Fluorouracil^®^ (5-FU) **I** as thymidylate synthase inhibitor^16^, Merbarone^®^ **II** as DNA topoisomerase II (topoII) catalytic inhibitor^17^, Ceritinib (LDK378) **III** as anaplastic lymphoma kinase (ALK) inhibitor^18^, Dasatinib **IV** as multi-targeted of Bcr-Abl and Src family kinases inhibitor apoptosis inducer^19^, Imatinib **V** as a receptor tyrosine kinase (TKI) inhibitor^20^, Ibrutinib (IBR) **VI** as Bruton’s tyrosine kinase (BTK) inhibitor^21^, Ruxolitinib (INC424) **VII** as Janus kinase (JAK) inhibitor^22^ and Nilotinib **VIII** as tyrosine kinase inhibitor and apoptosis inducer^23^ (Figure 1).

**Figure 2.**
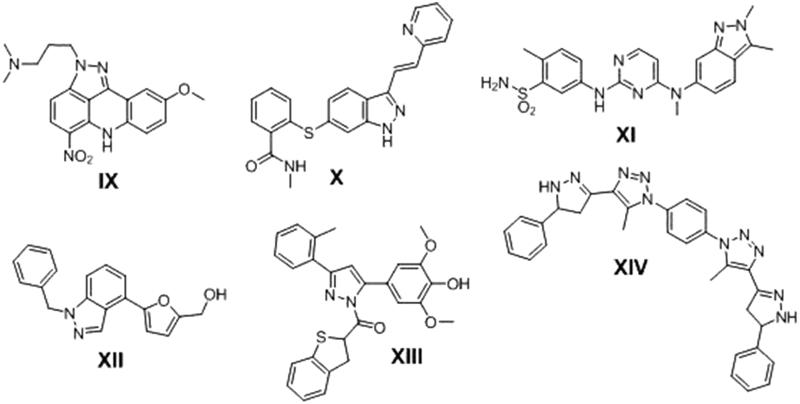
Biologically active anticancer compounds have pyrazoline ring.

Pyrazoline ring is another widely used heterocyclic nucleus in medicinal chemistry because of its wide spectrum of pharmacological activities i.e. anti-inflamatory^24^, antibacterial^25^, and antioxidant^26^ activities. Many pyrazoline derivatives was found to have anticancer activity against various human cancer types with different mode of action i.e. Pyrazoloacridine (PZA) **(IX)** ^27^, Axitinib (AG013736) **(X)** ^28^, Pazopanib (GW786034) **(XI)** ^29^ and 3–(5′-Hydroxymethyl-2′-furyl)-1-benzyl indazole (YC-1) **(XII)** ^30^. New pyrazoline derivatives **XIII** and **XIV** were reported as potential anticancer agents in hepatic HepG2 cancer cell line and induced HepG2 cells apoptosis ^31^ ^,^ ^32^ (Figure 2).

**Figure 3.**
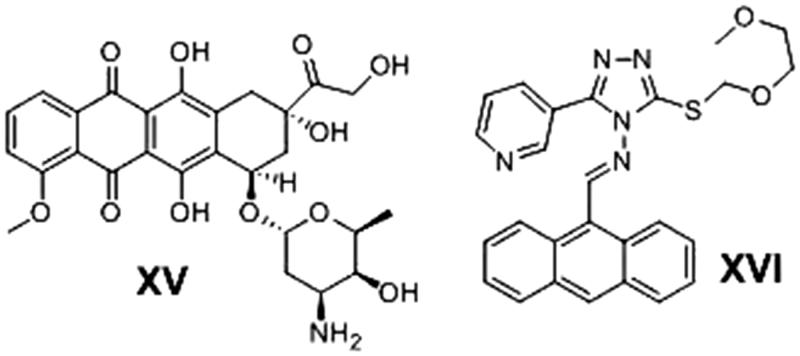
Examples of some anthracene-based anticancer agents.

Another organic moiety of high interest as anticancer pharmacophore is the anthracene nucleus. Several anthracene derivatives showed significant anticancer activity against a wide range of human tumour cell lines^33–35^ i.e. the well-known Doxorubicin (DOX) **XV,** is a Topoisomerase II inhibitor^36^ and anthracene-9-ylmethylene-[2-methoxyethoxymethylsulfanyl]-5-pyridin-3-yl- [1, 2, 4] triazole-4-amine (HL-37) **XVI** ^37^ (Figure 3).

**Figure 4.**
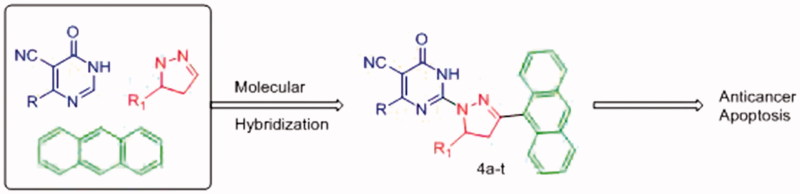
Design strategy of new pyrimidine hybrid compounds as anticancer agent.

The medicinal chemistry importance of the three pharmacophores, i.e. pyrimidine, pyrazoline and anthracene, encouraged us to design novel series of compounds hybridised with the three moieties. The hybridisation of these pharmacophores in a single molecule (Figure 4) is expected to result in a hybrid molecule that possesses anticancer activity. In this study, we report the design, synthesis and anticancer activity of a novel hybrids of pyrimidine derivatives (**4a-t)** bearing pyrazoline and anthracene pharmacophores against both human liver cancer cell lines (HepG2 and Huh-7) and normal fibroblast cells by resazurin assay. In addition, apoptosis and effects on caspase-3 and -7 activation against human liver cancer cell lines HepG2 and Huh-7 were also performed for the most active derivatives. Structure and activity relationship (SAR) and possible mechanisms of action of these compounds were also investigated.

## Materials and methods

### Instruments

All melting points were determined with Electro-thermal IA 9100 apparatus (Shimadzu, Tokyo, Japan) and were uncorrected. FT-IR spectra were recorded as potassium bromide pellets on a Perkin-Elmer 1650 spectrophotometer (PerkinElmer, Waltham, MA), Faculty of Science, Cairo University, Cairo, Egypt. ^1^HNMR and ^13^C-NMR spectra were recorded in DMSO-d_6_ on a Varian Mercury (300 MHz) spectrometer (Varian, Oxford, UK) and chemical shifts were given as ppm from TMS as internal reference (Faculty of Science, Cairo University, Cairo, Egypt). Mass spectra were recorded on 70 eV EI Ms-QP 1000 EX (Shimadzu, Tokyo, Japan), Faculty of Science, Cairo University, Cairo, Egypt. Microanalyses were performed using Vario, Elementar apparatus (Shimadzu, Tokyo, Japan), Organic Microanalysis Unit, Faculty of Science, Cairo University, Cairo, Egypt and the results were within the accepted range (0.40) of the calculated values. Column chromatography was performed on (Merck & Co., Kenilworth, NJ) silica gel 60 (particle size 0.06–0.20 mm).

### Chemistry

#### 6-Aryl-4-oxo-2-thioxo-1,2,3,4-tetrahydropyrimidine-5-carbonitriles (1a-e)

The titled compounds **1a-e** was synthesised according to the reported methods^38^.

#### 6-Aryl-2-hydrazinyl-4-oxo-1,4-dihydropyrimidine-5-carbonitriles (2a-e)

The titled compounds 2a^39^, 2b^40^ ^,^ ^41^, 2c^42^, 2d^43^, and 2e were synthesised according to the reported methods.

A mixture of **1a-d** (0.005 mol) and hydrazine hydrate (0.005 mol, 99%) in 30 mL ethanol was refluxed for 30 h, then cooled and poured on ice/water. The produced precipitate was filtered off, dried and crystallised from ethanol to give compounds **2a-e**.

##### 4-[4-(Dimethylamino)phenyl]-2-hydrazino-6-oxo-1,6-dihydropyrimidine-5-carbonitrile (2e)

Yellow solid, yield 60%, m.p. 214–216 °C. IR (KBr) *v* max (cm^−1^): 3366, 3295 (NH, NH_2_), 3069 (CH-Ar), 2959 (CH-sp3), 2209 (CN), 1689 (C=O).^1^H NMR (300 MHz, DMSO-d_6_) *δ*: 2.5 (s, 6H, CH_3_), 5.2 (s, 2H, NH_2_, D_2_O exchangeable), 7.38–7.57 (m, 4H, Ar-H), 10, 11 (s, 2H, 2NH, D_2_O exchangeable). ^13^C NMR (300 MHz, DMSO-d_6_): *δ* 43.6 (N-CH_3_), 90.4 (C-5 pyrimidine), 114.1–157.9 (aromatic Cs), 115.3 (CN), 159.5 (C-2 pyrimidine), 161.9(C=O), 175.7(C-6 pyrimidine). MS(EI): *m/z*: 270[M+] (12.3%),77 (100%). Anal. Calcd for C_13_H_14_N_6_O (270.29): C, 57.77; H, 5.22; N, 31.09; Found: C, 57.78; H, 5.32; N, 31.10.

#### (2E)-1–(9-anthryl)-3- substituted prop-2-en-1-ones (3a-d)

Compounds 3a, b, and c^44^ were synthesised according to the literature procedure.

##### General procedure for the preparation of compounds (3a-d)

A mixture of 9-acetylanthracene (0.01 mol) and the appropriate aldehyde (0.01 mol) in 50 mL 10% ethanolic KOH solution was stirred at room temperature for 24 h. The solution was cooled, poured on ice/water acidified with dil. HCl. The produced precipitate was filtered off, dried and crystallised from ethanol to give compounds **3a-d**.

##### (2E)-1–(9-anthryl)-3-(1H-indol-3-yl)prop-2-en-1-one (3d)

Brown solid, yield 85%, m.p. 170–172 °C. IR (KBr) *v* max (cm^−1^): 3177 (CH-Ar), 1665 (C=O), 1604 (C=C).^1^H NMR (300 MHz, DMSO-d_6_) *δ*: 6.6–8.7(m, 14H, Ar-H), 7.3 (d, 1H, *J* = 6.9 Hz,—CH=CH—), 7.5 (d, 1H, *J* = 19.2 Hz, —CH=CH—) 11.2 (s, 1H, NH, D_2_O exchangeable). ^13^C NMR (300 MHz, DMSO-d_6_) *δ*: 112.7–144.1 (aromatic Cs), 131.26, 140.8 (CH=CH), 189.7 (C=O). MS (EI): *m/z*: 347 [M+] (20%), 206 (100%). Anal. Calcd for C_25_H_17_NO (347.408): C, 86.43; H, 4.93; N, 4.03; O, 4.61; Found: C, 86.52; H, 5.03; N, 4.12; O, 4.70.

##### General procedure for the preparation of compounds (4a-t)

A mixture of compound **2a-e** (4 mmol), the appropriate propenone **3a-d** (4 mmol) and potassium hydroxide (0.2 g, 5 mmol) in absolute ethanol (30 mL) was refluxed for 72 h. The reaction mixture was poured on water, neutralised with 2 N hydrochloric acid and the residue was filtered off. The crude product obtained was crystallised from ethanol.

##### 2-[3-Anthracene-9-yl-5–(4-fluoro-phenyl)-4,5-dihydro-pyrazol-1-yl]-6-oxo-4-phenyl-1,6-dihydro-pyrimidine-5-carbonitrile (4a)

Yellow solid, yield 51%, m.p. 150–151 °C. IR (KBr) *v* max (cm^−1^): 3077 (CH-Ar), 2828 (CH-sp3), 2228 (CN), 1685 (C=O), 1603 (C=N).^ 1^HNMR (300 MHz, DMSO-d_6_) *δ*: 2.5–3.38 (dd, 2H, C_4_-Hpyrazoline), 4.50 (dd, 1H, *J* = 7.5 Hz, *J* = 13.8 Hz, C_5_-Hpyrazoline), 7–8.8 (m, 18H, Ar-H), 10.2 (s, 1H, NH, D_2_O exchangeable). ^13^C NMR (300 MHz, DMSO-d_6_): δ 41.7 (C-4 pyrazoline), 42.0 (C-5 pyrazoline), 91.7 (C-5 pyrimidine), 111.6–147.3 (aromatic Cs), 117.8 (CN), 152.4 (C-2 pyrazoline), 163.0 (C-2 pyrimidine), 168.1 (C=O), 175.7 (C, C-6 pyrimidine). MS (EI): *m/z*: 535 [M+] (8%), 178 (100). Anal. Calcd for C_34_H_22_FN_5_O (535.569): C, 76.25; H, 4.14; N, 13.08; Found: C, 76.29; H, 4.18; N, 13.17.

##### 2-[3-Anthracene-9-yl-5–(4-dimethylamino-phenyl)-4,5-dihydro-pyrazol-1-yl]-6-oxo-4-phenyl-1,6-dihydro-pyrimidine-5-carbonitrile (4b)

Yellowish brown crystals, yield 58%, m.p. 120–122 °C. IR (KBr) *v* max (cm^−1^): 3062 (CH-Ar), 2920 (CH-sp3), 2223(CN), 1681 (C=O), 1620 (C=N). ^1^H NMR (300 MHz, DMSO-d_6_) *δ*: 2.3, 2.4 (s, 6H, N-CH_3_), 2.5–3.5 (dd, 2H, C_4_-H pyrazoline), 5.2 (dd, 1H, *J* = 6.6 Hz, *J* = 17.8 Hz, C_5_-H pyrazoline), 7.1–8.9 (m, 18H, Ar-H), 10.1(s, 1H, NH, D_2_O exchangeable). ^13 ^C NMR (300 MHz, DMSO-d_6_): δ 41.0 (C-4 pyrazoline), 42.1 (C-5 pyrazoline), 43.0 (N-CH_3_), 91.2 (C-5 pyrimidine), 110.0–140.0 (aromatic Cs), 117.0 (CN), 152.0 (C-2 pyrazoline), 163.0 (C-2 pyrimidine), 168.0 (C=O), 175.0 (C, C-6 pyrimidine). MS (EI): *m/z*: 560 [M+] (4%), 439 (100%). Anal. Calcd for C_36_H_28_ N_6_O (560.647): C, 77.12; H, 5.03; N, 14.99; Found: C, 77.18; H, 5.14; N, 15.02.

##### 2-[3-Anthracene-9-yl-5–(3,4,5-trimethoxy-phenyl)-4,5-dihydro-pyrazol-1-yl]-6-oxo-4-phenyl-1,6-dihydro-pyrimidine-5-carbonitrile (4c)

Brown crystals, yield 60%, m.p. 110–112 °C. IR (KBr) *v* max (cm^−1^): 3060 (CH-Ar), 2922, 2850 (CH-sp3), 2222 (CN),1680 (C=O), 1618 (C=N). ^1^H NMR (300 MHz, DMSO-d_6_) *δ*: 2.6–3.2 (dd, 2H, C4-H pyrazoline), 5.4 (dd, 1H, *J* = 6.4 Hz, *J* = 17.5 Hz, C5-H pyrazoline), 3.3–3.5 (s, 9H, OCH_3_), 6.9–8.5 (m, 16H, Ar-H), 10.2 (s, 1H, NH, D_2_O exchangeable). ^13^C NMR (300 MHz, DMSO-d_6_): *δ* 41.2 (C-4 pyrazoline), 42.0 (C-5 pyrazoline), 56.2, 56.4 (OCH_3_),92.0 (C-5 pyrimidine), 111.0–142.0 (aromatic Cs), 117.0 (CN), 152.2 (C-2 pyrazoline), 163.0 (C-2 pyrimidine), 168.0 (C=O), 176.0 (C, C-6 pyrimidine). MS (EI): *m/z*: 607 [M+] (6%), 437 (100%). Anal. Calcd for C_37_H_29_N_5_O_4_ (607.657): C, 73.13; H, 4.81; N, 11.53; Found: C, 73.18; H, 4.90; N, 11.59.

##### 2-[3-Anthracene-9-yl-5-(1H-indol-3-yl)-4,5-dihydro-pyrazol-1-yl]-6-oxo-4-phenyl-1,6-dihydro-pyrimidine-5-carbonitrile(4d)

Yellow crystals, yield 59%, m.p. 135–137 °C. IR(KBr) *v* max (cm^−1^): 3062 (CH-Ar), 2852 (CH-sp3), 2220 (CN), 1682 (C=O), 1608 (C=N). ^1^HNMR (300 MHz, DMSO-d_6_) *δ*: 2.51–3.42 (dd, 2H, C4-H pyrazoline), 4.51 (dd, 1H, *J* = 3.3 Hz, *J* = 14.7 Hz, C5-H pyrazoline), 7.4–8.40 (m, 19 H, Ar-H), 10.2, 11.8 (s, 2H, NH, D_2_O exchangeable). ^13^C NMR (300 MHz, DMSO-d_6_): *δ* 41.5 (C-4 pyrazoline), 42.2 (C-5 pyrazoline), 91.2 (C-5 pyrimidine), 112.0–145.0 (aromatic Cs), 117 (CN), 152.0 (C-2 pyrazoline), 162.0 (C-2 pyrimidine), 168.0 (C=O), 170.0 (C, C-6 pyrimidine). MS (EI): *m/z*: 556 [M+] (3%), 419 (100%). Anal. Calcd for C_36_H_24_N_6_O (556.61536): C, 77.68; H, 4.35; N, 15.10; Found: C, 77.71; H, 4.41; N, 15.22.

##### 2-[3-Anthracene-9-yl-5–(4-fluoro-phenyl)-4,5-dihydro-pyrazol-1-yl]-4–(4-fluoro-phenyl)-6-oxo-1,6-dihydro-pyrimidine-5-carbonitrile (4e)

Yellowish brown crystals, yield 50%, m.p. 138–140 °C. IR (KBr) *v* max (cm^−1^): 3058 (CH-Ar), 2920, 2848 (CH-sp3), 2222(CN), 1672 (C=O), 1621 (C = N). ^1^H NMR (300 MHz, DMSO-d_6_) *δ*: 2.50–3.38 (dd, 2H, C4-H pyrazoline), 5.5 (dd, 1H, *J* = 3.6 Hz, *J* = 12.6 Hz, C5-H pyrazoline), 7.30–8.25 (m, 17H, Ar-H), 10.1 (s, 1H, NH, D_2_O exchangeable). ^13^C NMR (300 MHz, DMSO-d_6_): *δ* 40.2(C-4 pyrazoline), 42.2 (C-5 pyrazoline), 93.0 (C-5 pyrimidine), 103.0–150.0 (aromatic Cs), 115.0 (CN), 158.2 (C-2 pyrazoline), 164.1 (C-2 pyrimidine), 86.2(C=O), 172.1 (C, C-6 pyrimidine). MS (EI): *m/z*: 553 [M+] (8%), 95 (100%). Anal. Calcd for C_34_H_21_F_2_N_5_ O (553.56): C, 73.77; H, 3.82; N, 12.65; Found: C, 73.80; H, 3.90; N, 12.70.

##### 2-[3-Anthracene-9-yl-5–(4-dimethylamino-phenyl)-4,5-dihydro-pyrazol-1-yl]-4–(4-fluoro-phenyl)-6-oxo-1,6-dihydro-pyrimidine-5-carbonitrile (4f)

Brown crystals, yield 54%, m.p. 130–132 °C. IR (KBr) *v* max (cm^−1^): 3059 (CH-Ar), 2922, 2872 (CH-sp3), 2220 (CN), 1680 (C=O), 1614 (C=N). ^1^H NMR (300 MHz, DMSO-d_6_) *δ*: 2.3, 2.5 (s, 6H, N-CH_3_), 2.65–3.1 (dd, 2H, C4-H pyrazoline), 5.6 (dd, 1H, *J* = 11.8 Hz, *J* = 17.8 Hz, C5-H pyrazoline), 7–8.5 (m, 17 H, Ar-H), 10.0 (s, 1H, NH, D_2_O exchangeable). ^13^C NMR (300 MHz, DMSO-d_6_): *δ* 40.4 (C-4 pyrazoline), 43.0 (N-CH_3_), 42.4 (C-5 pyrazoline), 93.3 (C-5 pyrimidine), 104.0–147.0 (aromatic Cs), 115.0 (CN), 158.0 (C-2 pyrazoline), 164.0 (C-2 pyrimidine), 168.2 (C=O), 172.0 (C, C-6 pyrimidine). MS (EI): *m/z*: 578 [M+] (12%), 482 (100%). Anal. Calcd for C_36_H_27_FN_6_O (578.637): C, 74.72; H, 4.70; N, 14.52; Found: C, 74.83; H, 4.80; N, 14.60

##### 2-[3-Anthracene-9-yl-5–(3,4,5-trimethoxy-phenyl)-4,5-dihydro-pyrazol-1-yl]-4–(4-fluoro-phenyl)-6-oxo-1,6-dihydro-pyrimidine-5-carbonitrile (4g)

Brown crystals, yield 58%, m.p. 154–156 °C. IR (KBr) *v* max (cm^−1^): 3062 (CH-Ar), 2930, 2850 (CH-sp3), 2223 (CN), 1681 (C=O), 1620 (C=N). ^1^HNMR (300 MHz, DMSO-d_6_) *δ*: 2.62–3.2 (dd, 2H, C4-H pyrazoline), 5.7 (dd, 1H, *J* = 6.4 Hz, *J* = 11.8 Hz, C5-H pyrazoline), 3.4–3.6 (s, 9H, OCH_3_), 7.1–8.6 (m, 15H, Ar-H), 10.1 (s, 1H, NH, D_2_O exchangeable). ^13^C NMR (300 MHz, DMSO-d_6_): *δ* 40.2 (C-4 pyrazoline), 42.3 (C-5 pyrazoline), 56.2, 56.4 (OCH_3_), 93.2 (C-5 pyrimidine), 105.0–145.0 (aromatic Cs), 115.0 (CN), 158.4 (C-2 pyrazoline), 164.2 (C-2 pyrimidine), 168.1 (C=O), 172.4 (C, C-6 pyrimidine). MS (EI): *m/z*: 625 [M+] (12%), 108 (100%). Anal. Calcd for C_37_H_28_FN_5_O_4_ (625.647): C, 71.03; H, 4.51; N, 11.19; Found: C, 71.13; H, 4.60; N, 11.22.

##### 2-[3-Anthracene-9-yl-5-(1H-indol-3-yl)-4,5-dihydro-pyrazol-1-yl]-4–(4-fluoro-phenyl)-6-oxo-1,6-dihydro-pyrimidine-5-carbonitrile (4h)

Brown crystals, yield 59%, m.p. 143–145 °C. IR (KBr) *v* max (cm^−1^): 3177 (CH-Ar), 2893 (CH-sp3), 2226 (CN), 1680 (C=O), 1604 (C=N). ^1^H NMR (300 MHz, DMSO-d_6_) δ: 2.51–3.38 (dd, 2H, C4-H pyrazoline), 4.51 (dd, 1H, *J* = 7.5 Hz, *J* = 10.8 Hz, C5-H pyrazoline), 7.0–8.38 (m, 18H, Ar-H), 9.58, 10.20 (s, 2H, NH, D_2_O exchangeable). ^13^C NMR (300 MHz, DMSO-d_6_): *δ* 40.2 (C-4 pyrazoline), 42 0.0 (C-5 pyrazoline), 93.7 (C-5 pyrimidine), 103.6–152.0 (aromatic Cs), 115.2 (CN), 158.0 (C-2 pyrazoline), 164.0 (C-2 pyrimidine), 168.0 (C=O), 172.0 (C, C-6 pyrimidine). MS (EI): *m/z*: 574 [M+] (10%), 178 (100%). Anal. Calcd for C_36_H_23_FN_6_ O (574.605): C, 75.25; H, 4.03; N, 14.63; Found: C, 75.31; H, 4.15; N, 14.71.

##### 2-[3-Anthracene-9-yl-5–(4-fluoro-phenyl)-4,5-dihydro-pyrazol-1-yl]-4-(1H-indol-3-yl)-6-oxo-1,6-dihydro-pyrimidine-5-carbonitrile (4i)

A brown solid, yield 55%, m.p. 184–185 °C. IR (KBr) *v* max (cm^−1^): 3062 (CH-Ar), 2940, 2850 (CH-sp3), 2219 (CN), 1682 (C=O), 1601 (C=N). ^1^HNMR (300 MHz, DMSO-d_6_) *δ*: 2.95–4.1 (dd, 2H, C4-H pyrazoline), 4.9 (dd, 1H, *J* = 6.5 Hz, *J* = 17.7 Hz, C5-H pyrazoline), 7–8.5 (m, 18H, Ar-H), 10.1, 10.3 (s, 2H, NH, D_2_O exchangeable). ^13^C NMR (300 MHz, DMSO-d_6_): *δ* 41.4 (C-4 pyrazoline), 43.0 (C-5 pyrazoline), 93.1 (C-5 pyrimidine), 102.1–148.1 (aromatic Cs), 114.2 (CN), 152.4 (C-2 pyrazoline), 162.0 (C-2 pyrimidine), 165.0 (C=O), 178.0 (C, C-6 pyrimidine). MS (EI): *m/z*: 574 [M+] (3%), 117 (100%). Anal. Calcd for C_36_H_23_FN_6_O (574.605): C, 75.25; H, 4.03; N, 14.63; Found: C, 75.29; H, 4.15; N, 14.68.

##### 2-[3-Anthracene-9-yl-5–(4-dimethylamino-phenyl)-4,5-dihydro-pyrazol-1-yl]-4-(1H-indol-3-yl)-6-oxo-1,6-dihydro-pyrimidine-5-carbonitrile (4j)

A dark brown solid, yield 59%, m.p. 240–242 °C. IR (KBr) *v* max (cm^−1^): 3064 (CH-Ar), 2920, 2850 (CH-sp3), 2218 (CN), 1686 (C=O), 1598 (C=N). ^1^HNMR (300 MHz, DMSO-d_6_) *δ*: 2.3, 2.5 (s, 6H, N-CH_3_), 2.95–4.2 (dd, 2H, C4-H pyrazoline), 4.80 (dd, 1H, *J* = 6.3 Hz, *J* = 17.8 Hz, C5-H pyrazoline), 6.8–8.6 (m, 18H, Ar-H), 10, 10.2 (s, 2H, NH, D_2_O exchangeable). ^13^CNMR (300 MHz, DMSO-d_6_): *δ* 41.6 (C-4 pyrazoline), 43.0 (N-CH_3_), 43.2 (C-5 pyrazoline), 93.0 (C-5 pyrimidine), 105.0–145.0(aromatic Cs), 114.0 (CN), 152.4 (C-2 pyrazoline), 162.0 (C-2 pyrimidine), 164.5 (C=O), 177.0 (C, C-6 pyrimidine). MS (EI): *m/z*: 599 [M+] (6%), 478 (100%). Anal. Calcd for C_38_H_29_N_7_O (599.683): C, 76.11; H, 4.87; N, 16.35; Found: C, 76.18; H, 4.91; N, 16.40.

##### 2-[3-Anthracene-9-yl-5–(3,4,5-trimethoxy-phenyl)-4,5-dihydro-pyrazol-1-yl]-4-(1H-indol-3-yl)-6-oxo-1,6-dihydro-pyrimidine-5-carbonitrile (4k)

A brown solid, yield 61%, m.p. 228–230 °C. IR (KBr) *v* max (cm^−1^): 3344 (CH-Ar), 2959 (CH-sp3), 2213 (CN), 1686 (C=O), 1606 (C=N). ^1^H NMR (300 MHz, DMSO-d_6_) *δ*: 2.5–3.03 (dd, 2H, C4-H pyrazoline), 5.5 (dd, 1H, *J* = 6 Hz, *J* = 15.6 Hz, C5-H pyrazoline), 3.3 (s, 9H, OCH_3_), 6.76–8.12 (m, 16H, Ar-H), 9.6, 10.6 (s, 2H, NH, D_2_O exchangeable). ^13^C NMR (300 MHz, DMSO-d_6_): δ 41.6 (C-4 pyrazoline), 43.7 (C-5 pyrazoline), 55.5, 55.7 (OCH_3_), 93.0 (C-5 pyrimidine), 102.2–146.7 (aromatic Cs), 114.0 (CN), 152.0 (C-2 pyrazoline), 162.9 (C-2 pyrimidine), 164.7 (C = O), 178.0 (C, C-6 pyrimidine). MS (EI): *m/z*: 646 [M+] (9%), 77 (100%). Anal. Calcd for C_39_H_30_N_6_O_4_ (646.6933): C, 72.43; H, 4.68; N, 13.00; Found: C, 72.48; H, 4.70; N, 13.04.

##### 2-[3-Anthracene-9-yl-5-(1H-indol-3-yl)-4,5-dihydro-pyrazol-1-yl]-4-(1H-indol-3-yl)-6-oxo–1,6-dihydro-pyrimidine-5-carbonitrile (4l)

Reddish brown crystals, yield 62%, m.p. 220–222 °C. IR (KBr) *v* max (cm^−1^): 3070 (CH-Ar), 2920, 2850 (CH-sp3), 2222 (CN), 1686 (C=O), 1608 (C=N). ^1^H NMR (300 MHz, DMSO-d_6_) *δ*: 2.62–3.03 (dd, 2H, C4-H pyrazoline), 5.6 (dd, 1H, *J* = 3.6 Hz, *J* = 13.2 Hz, C5-H pyrazoline), 6.7–7.9 (m, 19H, Ar-H), 10, 11.6, 11.7 (s, 3H, NH, D_2_O exchangeable). ^13^CNMR (300 MHz, DMSO-d_6_): *δ* 41.4 (C-4 pyrazoline), 43.0 (C-5 pyrazoline), 93.0 (C-5 pyrimidine), 104.0 –144.2 (aromatic Cs), 114.0 (CN), 152.2 (C-2 pyrazoline), 162.4 (C-2 pyrimidine), 164.0 (C=O), 178.0 (C, C-6 pyrimidine). MS (EI): *m/z*: 595 [M+] (12%), 178 (100%). Anal. Calcd for C_38_H_25_N_7_O (595.6514): C, 76.62; H, 4.23; N, 16.46; Found: C, 76.68; H, 4.33; N, 16.51.

##### 2-[3-Anthracene-9-yl-5–(4-fluoro-phenyl)-4,5-dihydro-pyrazol-1-yl]-6-oxo-4–(3,4,5-trimethoxy-phenyl)-1,6-dihydro-pyrimidine-5-carbonitrile (4m)

Dark brown crystals, yield 58%, m.p. 148–150 °C. IR (KBr) *v* max (cm^−1^): 3348 (CH-Ar), 2953, 2850 (CH-sp3), 2212 (CN), 1684 (C=O), 1601 (C=N). ^1^H NMR (300 MHz, DMSO-d_6_) *δ*: 2.9–3.1 (dd, 2H, C4-H pyrazoline), 4.9 (dd, 1H, *J* = 6.6 Hz, *J* = 16.5 Hz, C5-H pyrazoline), 3.45 (9H, s, OCH_3_), 6.8–8.7 (m, 15H, Ar-H), 11.4 (s, 1H, NH, D_2_O exchangeable). ^13^C NMR (300 MHz, DMSO-d_6_): *δ* 41.5 (C-4 pyrazoline), 43.7 (C-5 pyrazoline), 56.2, 56.7 (OCH_3_), 95.7 (C-5 pyrimidine), 102.0–146.4 (aromatic Cs), 115.5 (CN), 152.4 (C-2 pyrazoline), 159.6 (C-2 pyrimidine), 168.5(C=O), 175.7 (C, C-6 pyrimidine). MS (EI): *m/z*: 625 [M+] (12%), 77 (100%). Anal. Calcd for C_37_H_28_FN_5_O_4_ (625.647): C, 71.03; H, 4.51; N, 11.19; Found: C, 71.14; H, 4.60; N, 11.20.

##### 2-[3-Anthracene-9-yl-5–(4-dimethylamino-phenyl)-4,5-dihydro-pyrazol-1-yl]-6-oxo-4–(3,4,5-trimethoxy-phenyl)-1,6-dihydro-pyrimidine-5-carbonitrile (4n)

Dark brown crystals, yield 60%, m.p. 170–172 °C. IR (KBr) *v* max (cm^−1^): 3061 (CH-Ar), 2922, 2850 (CH-sp3), 2222 (CN), 1686 (C=O), 1620(C=N). ^1^H NMR (300 MHz, DMSO-d_6_) *δ*: 2.5, 2.8 (s, 6H, N-CH_3_), 2.95–3.4 (dd, 2H, C4-H pyrazoline), 3.5 (s, 9H, OCH_3_), 5.3 (dd, 1H, *J* = 6.5 Hz, *J* = 17.9 Hz, C5-H pyrazoline), 6.7–8.8 (m, 15H, Ar-H), 11.1 (s, 1H, NH, D_2_O exchangeable). ^13^C NMR (300 MHz, DMSO-d_6_): *δ* 41.2 (C-4pyrazoline), 42.2 (C-5 pyrazoline), 43.0 (N-CH_3_), 56.3, 56.4 (OCH_3_), 95.7 (C-5 pyrimidine), 102.0–148.0 (aromatic Cs), 115.0 (CN), 152.2 (C-2 pyrazoline), 160.1 (C-2 pyrimidine), 168.5 (C=O), 175.4 (C, C-6 pyrimidine). MS (EI): *m/z*: 725 [M+] (4%), 108 (100%). Anal. Calcd for C_39_H_34_N_6_O_4_ (650.725.06): C, 71.98; H, 5.27; N, 12.91; Found: C, 72.01; H, 5.31; N,13.02.

##### 2-[3-Anthracene-9-yl-5–(3,4,5-trimethoxy-phenyl)-4,5-dihydro-pyrazol-1-yl]-6-oxo-4–(3,4,5-trimethoxy-phenyl)-1,6-dihydro-pyrimidine-5-carbonitrile (4o)

Brown crystals, yield 62%, m.p. 165–166 °C. IR (KBr) *v* max (cm^−1^): 3061 (CH-Ar), 2920, 2850 (CH-sp3), 2223(CN), 1680 (C=O), 1618 (C=N). ^1^HNMR (300 MHz, DMSO-d_6_) δ: 2.6–3.1 (dd, 2H, C4-H pyrazoline), 4.85(dd, 1H, *J* = 6.6 Hz, *J* = 18.2 Hz, C5-H pyrazoline), 3.3, 3.5 (s, 18H, OCH_3_), 6.5–8.5 (m, 13H, Ar-H), 10.3 (s,1H, NH, D_2_O exchangeable). ^13^C NMR (300 MHz, DMSO-d_6_): *δ* 41.2 (C-4 pyrazoline), 42.7 (C-5 pyrazoline), 56.2, 56.4, 56.5 (OCH_3_), 95.2 (C-5 pyrimidine), 102.0–147.0 (aromatic Cs), 115.0 (CN), 152.4 (C-2 pyrazoline), 160.2 (C-2 pyrimidine), 168.2 (C=O), 175.7 (C, C-6 pyrimidine). MS (EI): *m/z*: 697 [M+] (5%), 457 (100%). Anal. Calcd for C_40_H_35_N_5_O_7_ (697.7352): C, 68.86; H, 5.06; N, 10.04; Found: C, 68.91; H, 5.17; N, 10.15.

##### 2-[3-Anthracene-9-yl-5-(1H-indol-3-yl)-4,5-dihydro-pyrazol-1-yl]-6-oxo-4–(3,4,5-trimethoxy-phenyl)-1,6-dihydro-pyrimidine-5-carbonitrile (4p)

Brown crystals, yield 63%, m.p. 157–158 °C. IR (KBr) *v* max (cm^−1^): 3064 (CH-Ar), 2920, 2850 (CH-sp3), 2220 (CN), 1686 (C=O), 1618 (C=N). ^1^H NMR (300 MHz, DMSO-d_6_) *δ*: 2.51–4.03 (dd, 2H, C4-H pyrazoline), 5.51 (dd, 1H, *J* = 6.5 Hz, *J* = 17.2 Hz, C5-H pyrazoline), 3.5 (s, 9H, OCH_3_), 6.5–8.7 (m, 16H, Ar-H), 11.2, 11.3 (s, 2H, NH, D_2_O exchangeable). ^13^CNMR (300 MHz, DMSO-d_6_): *δ* 41.3 (C-4 pyrazoline), 42.4 (C-5 pyrazoline), 56.2, 56.7 (OCH_3_), 95.1 (C-5 pyrimidine), 102.0–146.0 (aromatic Cs), 115.0 (CN), 152.0 (C-2pyrazoline), 160.1 (C-2 pyrimi dine), 168.5 (C=O), 175.4 (C, C-6 pyrimidine). MS (EI): *m/z*: 644 [M+] (9%), 77 (100%). Anal. Calcd for C_40_H_32_N_6_O_3_ (644.720): C, 74.52; H, 5.00; N, 13.04; Found: C, 74.61; H, 5.12; N, 13.15.

##### 2-[3-Anthracene-9-yl-5–(4-fluoro-phenyl)-4,5-dihydro-pyrazol-1-yl]-4–(4-dimethylamino-phenyl)-6-oxo-1,6-dihydro-pyrimidine-5-carbonitrile (4q)

Reddish brown solid, yield 57%, m.p. 182–184 °C. IR (KBr) *v* max (cm^−1^): 3066 (CH-Ar), 2923 (CH-sp3), 2222 (CN), 1680 (C=O), 1602 (C=N). ^1^H NMR (300 MHz, DMSO-d_6_) δ: 2.5 (s, 6H, N-CH_3_), 2.9–3.36 (dd, 2H, C4-H pyrazoline), 5.50 (dd, 1H, *J* = 6.4 Hz, *J* = 18.2 Hz, C5-H pyrazoline), 6.7–8.8 (m, 17H, Ar-H), 10.2 (s, 1H, NH, D_2_O exchangeable). ^13^C NMR (300 MHz, DMSO-d_6_): *δ* 40.2 (C-4 pyrazoline), 43.2 (N-CH_3_), 55.2 (C-5 pyrazoline), 93.2 (C-5 pyrimidine), 104.0–142.0 (aromatic Cs), 114.0 (CN), 152.1(C-2 pyrazoline), 164.0 (C-2 pyrimidine), 160.0 (C=O), 170.0 (C, C-6 pyrimidine). MS (EI): *m/z*: 578 [M+] (12%), 178 (100%). Anal. Calcd for C_36_H_27_FN_6_O (578.637): C, 74.72; H, 4.70; N, 14.52; Found: C, 74.80; H, 4.78; N, 14.60.

##### 2-[3-Anthracene-9-yl-5–(4-dimethylamino-phenyl)-4,5-dihydro-pyrazol-1-yl]-4–(4-dimethylamino-phenyl)-6-oxo-1,6-dihydro-pyrimidine-5-carbonitrile (4r)

Brown crystals, yield 59%, m.p. 174–175 °C. IR (KBr) *v* max (cm^−1^): 3061 (CH-Ar), 2920, 2850 (CH-sp3), 2222 (CN), 1682 (C=O), 1602 (C=N). ^1^H NMR (300 MHz, DMSO-d_6_) *δ*: 2.3, 2.5 (s, 12H, N-CH_3_), 2.95–3.23 (dd, 2H, C4-H pyrazoline), 5.70 (dd, 1H, *J* = 6.3 Hz, *J* = 17.6 Hz, C5-H pyrazoline), 6.7–8.5 (m, 17H, Ar-H), 10.1 (s, 1H, NH, D_2_O exchangeable). ^13^C NMR (300 MHz, DMSO-d_6_): *δ* 40.1 (C-4 pyrazoline), 43.1 (N-CH_3_), 55.4 (C-5 pyrazoline), 93.2 (C-5 pyrimidine), 104.0–144.0 (aromatic Cs), 114.0 (CN), 152.0 (C-2 pyrazoline), 164.0 (C-2 pyrimidine), 160.0 (C=O), 170.2 (C, C-6 pyrimidine). MS (EI): *m/z*: 603 [M+] (8%), 77 (100%). Anal. Calcd for C_38_H_33_N_7_O (603.714): C, 75.60; H, 5.51; N, 16.24; Found: C, 75.67; H, 5.61; N, 16.30.

##### 2-[3-Anthracene-9-yl-5–(3,4,5-trimethoxy-phenyl)-4,5-dihydro-pyrazol-1-yl]-4–(4-dimethylamino-phenyl)-6-oxo-1,6-dihydro-pyrimidine-5-carbonitrile (4s)

A brown solid, yield 61%, m.p. 160–162 °C. IR (KBr) *v* max (cm^−1^): 3064 (CH-Ar), 2922, 2850 (CH-sp3), 2222 (CN), 1684 (C=O), 1604 (CN). ^1^H NMR (300 MHz, DMSO-d_6_) *δ*: 2.5 (s, 6H, N-CH_3_), 2.85–3.3 (dd, 2H, C4-H pyrazoline), 5.60 (dd, 1H, *J* = 6.6 Hz, *J* = 17.6 Hz, C5-H pyrazoline), 3.5 (s, 9H, OCH_3_), 6.6–8.4 (m, 15H, Ar-H), 10.3(s, 1H, NH, D_2_O exchangeable). ^13^C NMR (300 MHz, DMSO-d_6_): *δ* 40.0 (C-4 pyrazoline), 43.3 (N-CH_3_), 55.2 (C-5 pyrazoline), 56.2, 56.7 (OCH_3_), 93.2 (C-5 pyrimidine), 104.0–143.0 (aromatic Cs), 114.0 (CN), 152.2 (C-2 pyrazoline), 164.0 (C-2 pyrimidine), 160.0 (C=O), 170.0 (C, C-6 pyrimidine). MS (EI): *m/z*: 650 [M+] (9%), 480 (100%). Anal. Calcd for C_39_H_34_N_6_O_4_ (650.725): C, 71.98; H, 5.27; N, 12.91; Found: C, 72.01; H, 5.32; N, 13.00.

##### 2-[3-Anthracene-9-yl-5-(1H-indol-3-yl)-4,5-dihydro-pyrazol-1-yl]-4–(4-dimethylamino-phenyl)-6-oxo-1,6-dihydro-pyrimidine-5-carbonitrile (4t)

A brown solid, yield 62%, m.p. 190–191 °C. IR (KBr) *v* max (cm^−1^): 3333 (CH-Ar), 2944 (CH-sp3), 2211(CN), 1681 (C=O), 1600 (C=N). ^1^H NMR (300 MHz, DMSO-d_6_) *δ:* 2.49 (s, 6H, N-CH_3_), 2.56–3.33 (dd, 2H, C4-H pyrazoline), 5.49 (dd, 1H, *J* = 1.5 Hz, *J* = 18 Hz, C5-H pyrazoline), 7.4–8.5 (m, 19H, Ar-H), 9.3, 10.09 (s, 2H, NH, D_2_O exchangeable). ^13^C NMR (300 MHz, DMSO-d_6_): *δ* 40.2 (C-4 pyrazoline), 43.8 (N-CH_3_), 55.4 (C-5 pyrazoline), 93.9 (C-5 pyrimidine), 104.2–141.0 (aromatic Cs), 114.0 (CN), 152.0 (C-2 pyrazoline), 164.0 (C-2 pyrimidine), 159.7.0 (C=O), 170.0 (C, C-6 pyrimidine). MS (EI): *m/z*: 599 [M+] (8%), 117 (100%). Anal. Calcd for C_38_H_29_N_7_O (599.68316): C, 76.11; H, 4.87; N, 16.35; Found: C, 76.14; H, 4.94; N,16.50.

### *In vitro* cytotoxicity activity

All compounds **2a-e**, **3a-d**, and PPAD’s **4a-t** were subjected to a screening system for evaluation of their anticancer activity against cell line of human cancer, namely liver (HepG2 and Huh-7) cancer and normal fibroblast cells obtained from the German Cancer Research Center (DKFZ), following the Resazurin Cell Growth Inhibition Assay^45^ in comparison to the standard treatment using DOX (see Supplementary).

### Caspase-Glo 3/7 assay

The influence of our test samples on caspase 3/7 activity in liver cancer cells (HepG2 cells) was detected using Caspase-Glo 3/7 Assay kit (Promega, Madison, WI) as previously described^45^ (see Supplementary).

#### Data analysis

Results are represented as M ± SEM of at least three independent experiments. Data analysis has been done using graph pad prism and Sigma Plot 11 software.

### Molecular modelling procedure

The modelling experiment described in his study was performed by using the Discovery Studio version 4.5 (Accelrys Inc., San Diego, CA) software as previously described^13^. The required pdb coordinates were downloaded from the Brookhaven website (www.rcsb.org). The hydrogen atoms were then added to both the small molecule and the DNA structure. The atom and bond types as well as the protonation state for the small molecule and the binding site were checked and corrected when needed. Water molecules were deleted. This was followed by minimising the complex with the Discovery Studio (DS) force field by using the default parameters. The binding mode of the designed compound and that of the DOX in bound to part of the DNA structure will be discussed later.

## Results and discussion

### Chemistry

**Scheme 1.**
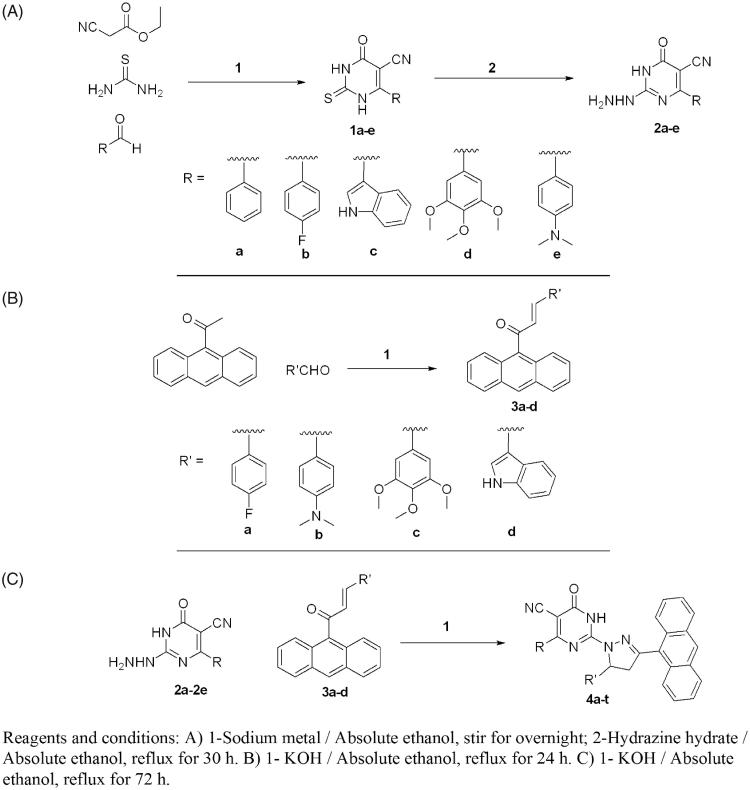
Synthesis of the designed compounds (**4a-t**).

The synthetic pathways adopted for the synthesis of the desired new compounds are illustrated in Scheme 1A–C. The starting 6-aryl-4-oxo-2-thioxo-1,2,3,4-tetrahydro pyrimidine-5-carbonitriles **(1a-e)** were synthesised from thiourea, ethyl cyanoacetate and the corresponding aldehydes in sodium ethoxide^38^ in a one pot reaction. The corresponding 2-hydrazino-6-oxo-4-phenyl/(4-substitutedphenyl)-1,6-dihydropyrimi-dine-5-carbonitriles **2a-e** were obtained through hydrazinolysis of the precursor thio derivatives^40^ (Scheme 1A). The (2*E*)-1–(9-anthryl)-3- substituted prop-2-en-1-ones derivatives **3a-d** were synthesised via a base-catalysed Claisen Schmidt condensation of the appropriate aldehyde and 9-acetylanthracene in 10% ethanolic KOH (Scheme 1B). Cyclocondensation reaction of the corresponding 2-hydrazinopyrimdine derivatives **2a-e** and the appropriate propenones **3a-d** in absolute ethanol in the presence of potassium hydroxide afforded the target compounds **4a-t** (Scheme 1C). The structures of the newly synthesised compounds were in good agreement with their IR, MS, ^1^H-NMR, ^13^C-NMR and elemental analyses data. The ^1^H NMR spectra of compounds **4a-t** showed the protons at C-4 and C-5 of the pyrazoline ring. Proton at C-4 appeared as doublet of doublet at *δ* 2.50–4.3 ppm and proton at C-5 appeared as doublet of doublet at *δ* 4.5–5.70 ppm. Other protons (N-CH_3_, OCH_3_, and aromatic Hs) were shown in their usual range. ^13^C-NMR showed the characteristic C-4 and C-5 carbon signals of the pyrazoline ring at *δ* 40– 41.7 and 42– 55.4 ppm, respectively, in addition to the other signals for the carbons of the target compounds (c.f. Experimental section).

### Anticancer evaluation

**Figure 5.**
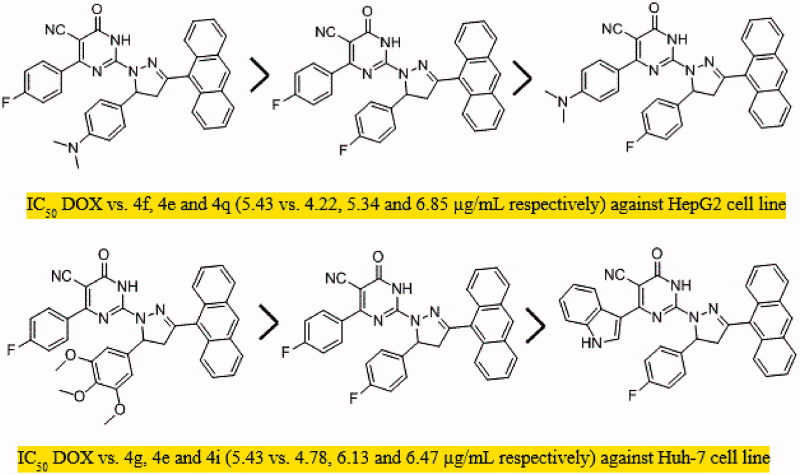
The most potent pyrimidine derivatives against HepG2 cell line and Huh-7 cell line, respectively.

This study presents the synthesis and antiproliferative activity of compounds having pyrimidine, pyrazoline, and anthracene pharmacophores. All compounds **2a-e**, **3a-d,** and PPAD’s **4a-t** were investigated for their *in vitro* anti-HCC activity against HepG2, Huh-7 cell lines, and normal fibroblast cells and the anticancer drug DOX was used as standard treatment. The cytotoxic activities of tested compounds were measured as IC_50_ (in μg/mL) value (the dose that reduces cell growth to 50%) (Table 1). The results showed that the tested compounds exhibited good to moderate anti-proliferative activities against the tested cell lines compared to normal cells. Compounds **2a-e**, **3a-d** were found to show moderate activity against the two cell lines. Regarding the activity of PPAD’s **4a-t** against HepG2 cell line, the results showed that compound **4f** (IC_50_=4.2 μg/mL which is equivalent to 7.2 μM) possessed the highest degree of cytotoxicity**, 4e** (IC_50_=5.34 μg/mL which is equivalent to 9.6 μM) was almost equipotent to DOX (IC_50_=5.43 μg/mL which is equivalent to 10.1 μM) and **4q** (IC_50_=6.85 μg/mL which is equivalent to 11.8 μM) was quite less potent than DOX (Figure 5). The anticancer activity of the tested PPAD’s against HepG2 cell line had the following descending order: **(4f > 4e > 4q > 4g > 4h > 4m > 4r > 4s> 4t > 4i > 4n > 4o > 4p >4a> 4j > 4k > 4l > 4b > 4c >4d)**.

**Table 1.** IC_50_ values in μg/mL for cytotoxic activity of the compounds against hepatocellular carcinoma cell lines and fibroblast cells.

Compounds	*Cytotoxic activity IC**_50_** (μg/mL)		
	HepG2 cell line	Huh-7 cell line	Fibroblast
**2a**	20.8 ± 1.6	21.7 ± 1.18	40.01 ± 1.24
**2b**	22.2 ± 2.20	21.6 ± 2.19	40.58 ± 2.45
**2c**	20.2 ± 1.88	21.3 ± 2.37	40.23 ± 2.13
**2d**	20.6 ± 2.14	21.8 ± 3.18	40.13 ± 1.37
**2e**	20.1 ± 3.00	21 ± 4.12	40.42 ± 1.89
**3a**	22.2 ± 2.45	21.8 ± 1.24	40.56 ± 2.48
**3b**	21.4 ± 2.76	21.00 ± 1.12	40.71 ± 2.34
**3c**	21.5 ± 2.54	21.5 ± 4.13	40.82 ± 4.51
**3d**	22.5 ± 2.80	22.6 ± 3.30	40.81 ± 4.72
**4a**	11.34 ± 2.11	16.75 ± 3.45	32.31 ± 5.22
**4b**	15.31 ± 4.21	10.95 ± 2.14	33.02 ± 6.51
**4c**	15.32 ± 4.22	11.33 ± 1.09	29.32 ± 3.51
**4d**	17.24 ± 4.04	8.42 ± 1.04	21.51 ± 4.07
**4e**	5.34 ± 0.21	6.13 ± 1.01	24.12 ± 4.63
**4f**	4.22 ± 0.94	8.45 ± 0.75	33.14 ± 6.52
**4g**	7.22 ± 1.85	4.78 ± 0.24	25.32 ± 4.87
**4h**	7.45 ± 1.11	9.77 ± 1.48	23.52 ± 4.21
**4i**	9.54 ± 1.24	6.47 ± 0.41	31.42 ± 2.07
**4j**	12.18 ± 2.53	13.24 ± 3.05	34.14 ± 5.17
**4k**	14.13 ± 3.01	13.52 ± 2.32	27.04 ± 4.12
**4l**	14.63 ± 2.12	12.44 ± 1.75	20.23 ± 3.18
**4m**	8.12 ± 1.82	7.50 ± 0.98	24.13 ± 4.34
**4n**	9.66 ± 1.31	9.54 ± 2.16	18.57 ± 3.17
**4o**	10.19 ± 1.02	14.52 ± 1.24	28.05 ± 3.31
**4p**	11.21 ± 1.2	8.14 ± 1.65	18.24 ± 4.02
**4q**	6.85 ± 0.85	8.66 ± 1.52	34.31 ± 6.42
**4r**	8.44 ± 1.10	7.61 ± 0.21	25.24 ± 5.24
**4s**	8.66 ± 1.44	11.28 ± 0.61	20.31 ± 4.15
**4t**	9.21 ± 1.05	7.42 ± 1.48	23.51 ± 3.82
**Doxorubicin**	5.43 ± 0.24	6.40 ± 0.43	24.11 ± 3.53

*Three independent experiments were performed for each concentration.

To shed the light on the SAR of these series of compound. The data suggest that the promising compounds emerging were those substituted with fluorine atom (-F) in both R and R^’^ such as **4e** or -F and N, N(CH_3_)_2_ such as **4f** and **4q**. Compounds substituted with both (R) and (R^’^) were more effective than those having only R^’^ substituent as in **4b-d** against HepG2 cell line. On the other hand, testing the compounds against Huh-7 cell line, all the tested PPAD’s **4a-s** showed anticancer activities with IC_50_ values ranging from 4.78 to 16.75 μg/mL. Interestingly, compounds **4e** and **4g** (IC_50_=6.13 which is equivalent to 11.1 μM and 4.78 μg/mL which is equivalent to 7.6 μM, respectively) showed activity against Huh-7 cell line higher than DOX. Compound **4i** (IC_50_=6.47 μg/mL which is equivalent to 11.25 μM) showed activity comparable to DOX (Figure 5). However, compounds **4m**, **4r**, **4t**, **4f**, **4p,** and **4q** had significant activity (IC_50_=7.50, 7.61, 7.42, 8.45, 8.14, and 8.66 μg/mL, respectively). The activity of the tested compounds against Huh-7cell line had the following descending order: **(4g > 4e >4i > 4t > 4m > 4r > 4p> 4d >4f > 4q > 4n> 4h >4b > 4s> 4c > 4l > 4j > 4k > 4o> 4a)**.

**Figure 6.**
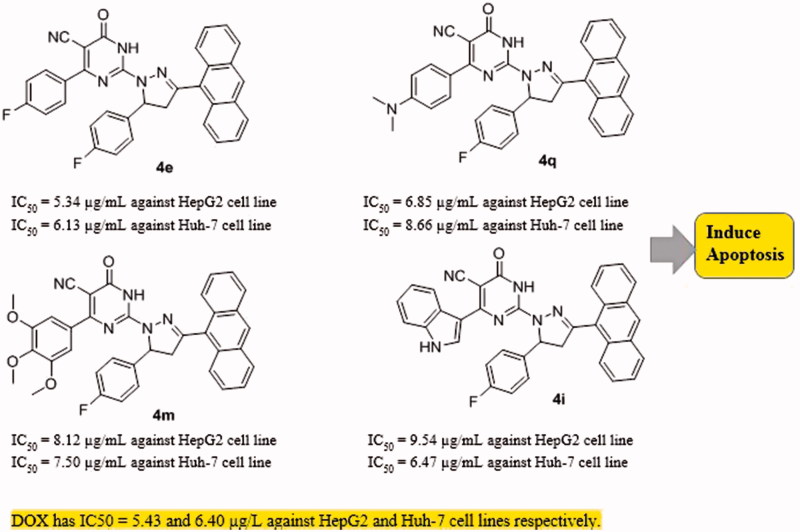
The most potent PPADs as anticancer agents.

**Figure 7.**
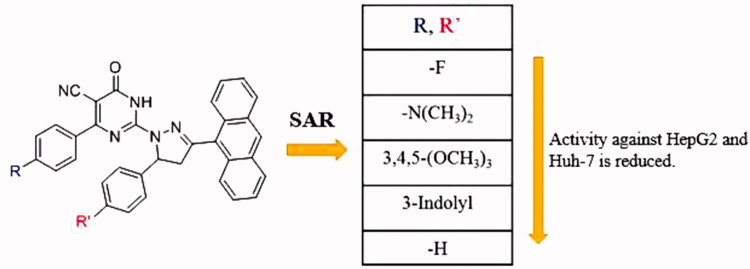
Structure activity relationship of the pyrimidine derivatives.

The data presented in this study revealed that all the tested PPAD’s **4a-t** had broad spectrum anticancer activity against all screened HCC cell lines compared to normal cells. Some of our PPAD’s exhibited potent antiproliferative activity against HepG2 and Huh-7 cell lines. The cytotoxicity of all compounds was more prominent on cancer cells compared to normal ones (Table 1). These results suggest that pyrimidine pyrazoline-anthracene backbone is an interesting anticancer pharmacophore. Moreover, some PPAD’s were even more potent than the standard drug Dox. The most potent compound in this study was **4e**. This could be explained by the electronegative effect of the fluorine atom on the pyrimidine and pyrazoline -anthracene backbone. PPAD’s **4e, 4i, 4m,** and **4q** were the most promising anticancer agents against all the tested cell lines (Figure 6). Interestingly, PPAD’s **4e** showed high potency against HepG2 and Huh-7 cell lines. Regarding the structure SAR of compounds (Figure 7), there was a consistent relation between the lipophilicity and/or electronic property of the substituent groups R and R^’^ groups and the antiproliferative activity. Concerning the nature of substituent groups R and R^’^, the order of activity of tested compounds against tested cell lines was the following: (F > N(CH_3_)_2_ >3,4,5-(OCH_3_)_3_ > 3-Indolyl > H). The introduction of a more electronegative and a lipophilic substituent to phenyl rings of the pyrimidine and pyrazoline enhance potency against HepG2 and Huh-7 cancer cell lines as shown in the fluorine substituted PPAD’s **4e**. However, a drop in potency against the same cell lines was observed with unsubstituted phenyl ring or 3-indolyl substituted PPAD’s as in **4a-d**. The sensitivity of the tested cell lines to PPAD’s **4a-t** was in the following descending order: (HepG2 > Huh-7).

### Caspase 3/7 assay

**Figure 8.**
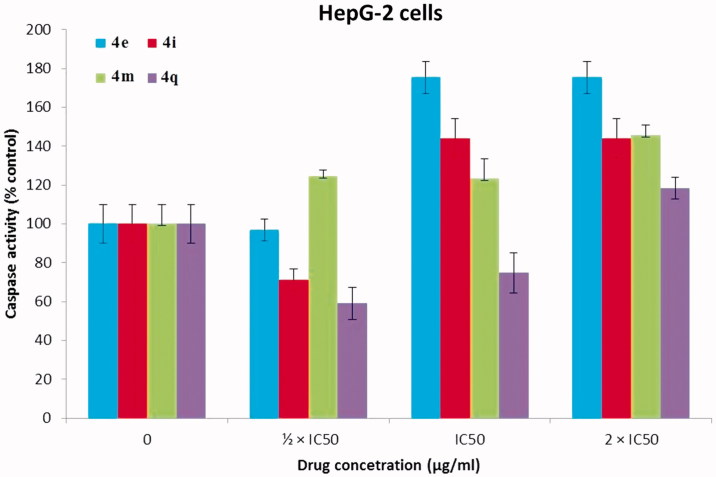
Caspase 3/7 assay results of PPADs **4e**, **4i**, **4m,** and **4q** against HepG2 cell line (24 h incubation). The results were significant; *p* < .05, *n* = 3.

**Figure 9.**
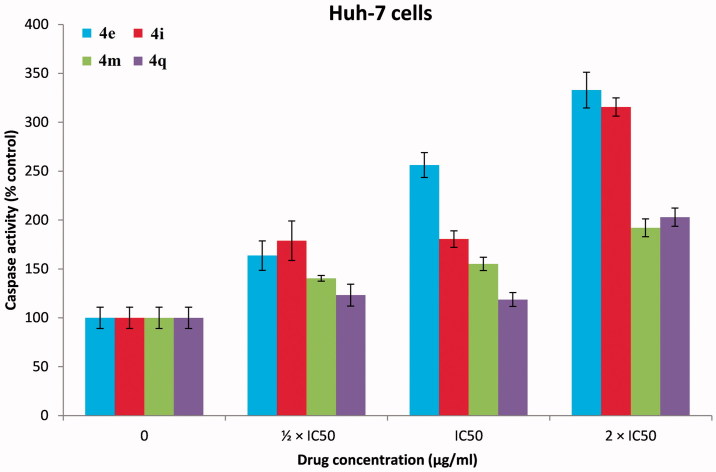
Caspase 3/7 assay results of PPADs **4e**, **4i**, **4m,** and **4q** against Huh-7 cell line (24 h incubation). The results were significant; *p* < .05, *n* = 3.

The caspases are a family of endoproteases that provide critical links in cell regulatory networks through controlling inflammation and cell death (apoptosis). Caspase-3 and caspase-7 cleave proteins involved in programmed cell death events^46^. It is well established that the induction of the apoptotic cascade is one of the main mechanisms of most of the currently available anticancer agents^45^ ^,^ ^47^ ^,^ ^48^. To determine the mechanism involved in the antitumor activity of our PPADs demonstrated above whether is a result of apoptosis or not, Caspase-Glo 3/7 assay was performed^47^. HepG-2 (Figure 8) and Huh-7 cell lines (Figure 9) were treated with the target PPAD’s samples or DMSO (solvent control). Figures 8 and 9 show that PPADs **4e, 4i, 4m,** and **4q** stimulated caspase activity in both cell lines at all tested concentrations and caused significant increase in activation of caspase 3/7 in a dose-dependent manner. These results suggest that our compounds induced apoptosis is, in part, due to activation of caspses3/7 and apoptosis is may be the main mechanism of action. Moreover, the apoptosis induced by the tested target compounds on Huh-7 cell line was greater than its effect on HepG2 cell line.

### Molecular modelling

**Figure 10.**
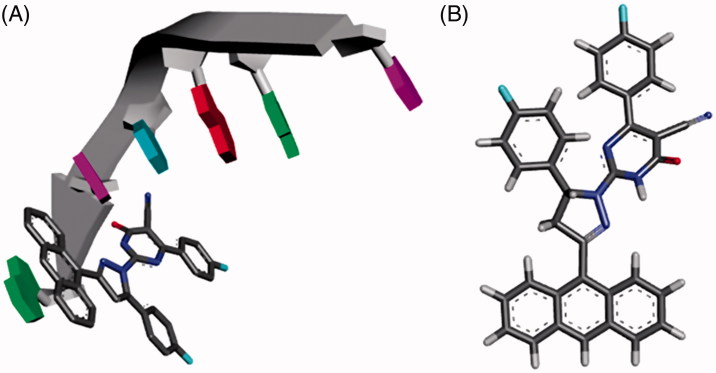
**(**A) The modeled structure of compound 4e predicting that compound **4e** will form a covalent crosslink to DNA using the anthracene ring while the rest of the compound is in the minor groove like the crystal structure of DOX (pdb:1D12); (B) 3 D structure of compound **4e**.

One possible reason for the potent anticancer activity of the designed compounds is the presence of the planar aromatic tricyclic (Anthracene) ring. The anthracene containing compounds, such as DOX or the designed compounds **4a-t**, are hypothesised to function primarily at the DNA level by blocking the replication and transcription processes. The binding to DNA structure is generally hypothesised to be essential for the cytotoxic activity of these compounds. To predict and understand the possible binding mode and the respective interactions of the designed compounds with the cell DNA structure, the co-crystal structure of DOX-DNA sequence d(CGATCG) complex (PDB: 1D12) was used^49^. As shown in Figure 10, the anthracene planar chromophore of compound **4e** is intercalated with the DNA helix. While the rest of the structure is directed towards the minor groove of DNA forming additional van der Waals interactions.

## Conclusions

A novel series of pyrimidine pyrazoline-anthracene hybrids **4a-t** were designed and synthesised via a cyclocondensation reaction of 2-hydrazinopyrimdine derivatives **2a-e** and the appropriate chalcones **3a-d** and their spectral and elemental analyses proved chemical structures. The antiproliferative activity of all synthesised compounds was screened against both HCC cell lines (HepG2 and Huh-7) and normal fibroblast cells, using DOX as reference. PPAD’s **4a-t** had broad spectrum anticancer activity against two HCC cell lines compared to normal cells. Compound **4f** possessed the most potent cytotoxicity against HepG2 cell line. In addition, PPAD’s **4e** and **4g** showed decent activity against Huh-7 cell line higher than DOX. Compound **4e** showed high potency against HepG2 and Huh-7 cell lines. PPAD’s **4e, 4i, 4m,** and **4q** were the most promising anticancer agents against all the tested cell lines. Further studies on the mechanism of action demonstrated that these compounds induce apoptosis in HepG2 and Huh-7 cell lines through significant activation of caspase 3/7 at all tested concentrations. The molecular modelling study performed suggested another possible mechanism of action for these compounds. Similar to the DOX, the presence of the tricyclic planar anthracene chromophore could intercalate with the DNA helix of the cancer cell. While the rest of the structure will be directed towards the minor groove of DNA forming additional van der Waals interactions. In conclusion, the designed pyrimidine pyrazoline-anthracene scaffold is an interesting anticancer pharmacophore and considered as novel lead scaffold for any future optimisation.

## Supplementary Material

Supplemental Material

## References

[1] Siegel R, DeSantis C, Virgo K, et al. Cancer treatment and survivorship statistics, 2012. CA Cancer J Clin 2012;62:220–41. 22700443 10.3322/caac.21149

[2] Siegel RL, Miller KD, Jemal A Cancer statistics, 2018. CA Cancer J Clin 2018;68:7–30. 29313949 10.3322/caac.21442

[3] Tannus RK, Almeida-Carvalho SR, Loureiro-Matos CA, et al. Evaluation of survival of patients with hepatocellular carcinoma: a comparative analysis of prognostic systems. PLoS One 2018;13:e0194922. 29617435 10.1371/journal.pone.0194922 PMC5884519

[4] Zhuang H, Jiang W, Cheng W, et al. Down-regulation of HSP27 sensitizes TRAIL-resistant tumor cell to TRAIL-induced apoptosis. Lung Cancer 2010;68:27–38. 19540014 10.1016/j.lungcan.2009.05.014

[5] Jin K, Yin H, De Clercq E, et al. Discovery of biphenyl-substituted diarylpyrimidines as non-nucleoside reverse transcriptase inhibitors with high potency against wild-type and mutant HIV-1. Eur J Med Chem 2018;145:726–34. 29353724 10.1016/j.ejmech.2018.01.016

[6] Bai S, Liu S, Zhu Y, Wu Q Asymmetric synthesis and antiviral activity of novel chiral amino-pyrimidine derivatives. Tetrahedron Lett 2018;59:3179–83.

[7] Madhu Sekhar M, Nagarjuna U, Padmavathi V, et al. Synthesis and antimicrobial activity of pyrimidinyl 1,3,4-oxadiazoles, 1,3,4-thiadiazoles and 1,2,4-triazoles. Eur J Med Chem 2018;145:1–10. 29310025 10.1016/j.ejmech.2017.12.067

[8] Rizk SA, El-Naggar AM, El-Badawy AA Synthesis, spectroscopic characterization and computational chemical study of 5-cyano-2-thiouracil derivatives as potential antimicrobial agents. J Mol Struct 2018;1155:720–33.

[9] Saleeb M, Sundin C, Aglar O, et al. Structure-activity relationships for inhibitors of *Pseudomonas* aeruginosa exoenzyme S ADP-ribosyltransferase activity. Eur J Med Chem 2018;143:568–76. 29207339 10.1016/j.ejmech.2017.11.036

[10] Borik RM, Fawzy NM, Abu-Bakr SM, Aly MS Design, synthesis, anticancer evaluation and docking studies of novel heterocyclic derivatives obtained via reactions Involving curcumin. Molecules 2018;23:1398. 10.3390/molecules23061398 PMC6099980 29890691

[11] Abdelgawad MA, Bakr RB, Azouz AA Novel pyrimidine-pyridine hybrids: synthesis, cyclooxygenase inhibition, anti-inflammatory activity and ulcerogenic liability. Bioorg Chem 2018;77:339–48. 29421710 10.1016/j.bioorg.2018.01.028

[12] Mohamed MS, Youns MM, Ahmed NM Synthesis, antimicrobial, antioxidant activities of novel 6-aryl-5-cyano thiouracil derivatives. Eur J Med Chem 2013;69:591–600. 24095752 10.1016/j.ejmech.2013.08.032

[13] Awad SM, Zohny YM, Ali SA, et al. Design, synthesis, molecular modeling, and biological evaluation of novel thiouracil derivatives as potential antithyroid agents. Molecules 2018;23:2913. 10.3390/molecules23112913 PMC6278332 30413058

[14] Sahu M, Siddiqui N, Sharma V, Wakode S 5,6-Dihydropyrimidine-1(2H)-carbothioamides: synthesis, in vitro GABA-AT screening, anticonvulsant activity and molecular modelling study. Bioorg Chem 2018;77:56–67. 29331765 10.1016/j.bioorg.2017.12.031

[15] Jha V, Bhadoriya KS Synthesis, pharmacological evaluation and molecular docking studies of pyrimidinedione based DPP-4 inhibitors as antidiabetic agents. J Mol Struct 2018;1158:96–105.

[16] Santi DV, McHenry CS, Sommer H Mechanism of interaction of thymidylate synthetase with 5-fluorodeoxyuridylate. Biochemistry 1974;13:471–81. 4203910 10.1021/bi00700a012

[17] Pastor N, Dominguez I, Orta ML, et al. The DNA topoisomerase II catalytic inhibitor merbarone is genotoxic and induces endoreduplication. Mutat Res 2012;738–739:45–51. 10.1016/j.mrfmmm.2012.07.005 22921906

[18] Cooper MR, Chim H, Chan H, Durand C Ceritinib: a new tyrosine kinase inhibitor for non-small-cell lung cancer. Ann Pharmacother 2015;49:107–12. 25258420 10.1177/1060028014553619

[19] Chan WY, Lau PM, Yeung KW, Kong SK The second-generation tyrosine kinase inhibitor dasatinib induced eryptosis in human erythrocytes-An in vitro study. Toxicol Lett 2018;295:10–21. 29803841 10.1016/j.toxlet.2018.05.030

[20] El-Mezayen NS, El-Hadidy WF, El-Refaie WM, et al. Hepatic stellate cell-targeted imatinib nanomedicine versus conventional imatinib: a novel strategy with potent efficacy in experimental liver fibrosis. J Control Release 2017;266:226–37. 28965860 10.1016/j.jconrel.2017.09.035

[21] Tang B, Tang P, He J, et al. Characterization of the binding of a novel antitumor drug ibrutinib with human serum albumin: insights from spectroscopic, calorimetric and docking studies. J Photochem Photobiol B Biol 2018;184:18–26. 10.1016/j.jphotobiol.2018.05.008 29777941

[22] Wilson GS, Tian A, Hebbard L, et al. Tumoricidal effects of the JAK inhibitor Ruxolitinib (INC424) on hepatocellular carcinoma in vitro. Cancer Lett 2013;341:224–30. 23941832 10.1016/j.canlet.2013.08.009

[23] Shaker ME, Ghani A, Shiha GE, et al. Nilotinib induces apoptosis and autophagic cell death of activated hepatic stellate cells via inhibition of histone deacetylases. Biochim Biophys Acta 2013;1833:1992–2003. 23499874 10.1016/j.bbamcr.2013.02.033 PMC5410771

[24] He J, Ma L, Wei Z, et al. Synthesis and biological evaluation of novel pyrazoline derivatives as potent anti-inflammatory agents. Bioorg Med Chem Lett 2015;25:2429–33. 25881822 10.1016/j.bmcl.2015.03.087

[25] Shubhalaxmi Pathak L, Ananda K, Bhat KS Synthesis of focused library of novel aryloxyacids and pyrazoline derivatives: molecular docking studies and antimicrobial investigation. Cogent Chem 2016;2:1141388.

[26] P. James J, Ishwar Bhat K, More UA, Joshi SD Design, synthesis, molecular modeling, and ADMET studies of some pyrazoline derivatives as shikimate kinase inhibitors. Med Chem Res 2018;27:546–59.

[27] Ramaswamy B, Mrozek E, Kuebler JP, et al. Phase II trial of pyrazoloacridine (NSC#366140) in patients with metastatic breast cancer. Invest New Drugs 2011;29:347–51. 19844661 10.1007/s10637-009-9338-1 PMC3486428

[28] Ranieri G, Mammi M, Donato Di Paola E, et al. Pazopanib a tyrosine kinase inhibitor with strong anti-angiogenetic activity: a new treatment for metastatic soft tissue sarcoma. Crit Rev Oncol Hematol 2014;89:322–9. 24041629 10.1016/j.critrevonc.2013.08.012

[29] Na JI, Na JY, Choi WY, et al. The HIF-1 inhibitor YC-1 decreases reactive astrocyte formation in a rodent ischemia model. Am J Transl Res 2015;7:751–60. 26064442 PMC4455349

[30] George DJ Phase 2 studies of sunitinib and AG013736 in patients with cytokine-refractory renal cell carcinoma. Clin Cancer Res 2007;13:753s–7s. 17255305 10.1158/1078-0432.CCR-06-2044

[31] Abd-Rabou AA, Abdel-Wahab BF, Bekheit MS Synthesis, molecular docking, and evaluation of novel bivalent pyrazolinyl-1,2,3-triazoles as potential VEGFR TK inhibitors and anti-cancer agents. Chem Pap 2018;72:2225–37.

[32] Xu W, Pan Y, Wang H, et al. Synthesis and evaluation of new pyrazoline derivatives as potential anticancer agents in hepG-2 cell line. Molecules 2017;22:pii: E467. 28300751 10.3390/molecules22030467 PMC6155299

[33] Sangthong S, Ha H, Teerawattananon T, et al. Overcoming doxorubicin-resistance in the NCI/ADR-RES model cancer cell line by novel anthracene-9,10-dione derivatives. Bioorg Med Chem Lett 2013;23:6156–60. 24095089 10.1016/j.bmcl.2013.09.004

[34] Kraicheva I, Vodenicharova E, Shenkov S, et al. Synthesis, characterization, antitumor activity and safety testing of novel polyphosphoesters bearing anthracene-derived aminophosphonate units. Bioorg Med Chem 2014;22:874–82. 24365391 10.1016/j.bmc.2013.12.001

[35] Pavitha P, Prashanth J, Ramu G, et al. Synthesis, structural, spectroscopic, anti-cancer and molecular docking studies on novel 2-[(Anthracene-9-ylmethylene) amino]-2-methylpropane-1,3-diol using XRD, FTIR, NMR, UV–Vis spectra and DFT. J Mol Struct 2017;1147:406–26.

[36] Cutts SM, Nudelman A, Rephaeli A, Phillips DR The power and potential of doxorubicin-DNA adducts. IUBMB Life 2005;57:73–81. 16036566 10.1080/15216540500079093

[37] Xie SQ, Zhang ZQ, Hu GQ, et al. HL-37, a novel anthracene derivative, induces Ca^(2+)^-mediated apoptosis in human breast cancer cells. Toxicology 2008;254:68–74. 18948164 10.1016/j.tox.2008.09.021

[38] Mohamed MS, Awad SM, Ahmed NM Synthesis and antimicrobial evaluation of some 6-aryl-5-cyano-2-thiouracil derivatives. Acta Pharm 2011;61:171–85. 21684845 10.2478/v10007-011-0019-1

[39] Marella A, Akhter M, Shaquiquzzaman M, et al. Synthesis, 3D-QSAR and docking studies of pyrimidine nitrile-pyrazoline: a novel class of hybrid antimalarial agents. Med Chem Res 2015;24:1018–37.

[40] Taher AT, Helwa AA Novel pyrimidinone derivatives: synthesis, antitumor and antimicrobial evaluation. Chem Pharm Bull (Tokyo) 2012;60:521–30. 22466736 10.1248/cpb.60.521

[41] Aqlan FMS, Makki MST, Abdel-Rahman RM Synthesis, spectroscopic studies of fluorinated pyrimido-1,2,4-triazines: protective effect against some plant pathogenic fungi. J Heterocycl Chem 2016;53:1310–7.

[42] Mohamed MS, Awad SM, Ahmed NM Synthesis and antimicrobial activities of new indolyl-pyrimidine derivatives. J Appl Pharm Sci 2011;1:76–80.

[43] Mohamed MS, Ahmed NM Synthesis, characterization and study of antimicrobial activity of some novel 5-cyano-2-thiouracil derivatives. Int J Pharm Sci 2014;4:591–600.

[44] Prabhakar M, Merry T, Tsurho S, et al. Synthesis, characterization and biological evaluation of 9-anthracenyl chalcones as anti-cancer agents. J Chem Pharm Res 2017;9:185–92.

[45] Nasr T, Bondock S, Youns M Anticancer activity of new coumarin substituted hydrazide-hydrazone derivatives. Eur J Med Chem 2014;76:539–48. 24607878 10.1016/j.ejmech.2014.02.026

[46] Riedl SJ, Shi Y Molecular mechanisms of caspase regulation during apoptosis. Nat Rev Mol Cell Biol 2004;5:897–907. 15520809 10.1038/nrm1496

[47] Abdallah MA, Gomha SM, Morad MA, Elaasser MM Synthesis of pyridotriazolopyrimidines as antitumor agents. J Heterocycl Chem 2017;54:1242–51.

[48] Abbas I, Gomha S, El-Neairy M, et al. Synthesis and biological evaluation of novel fused triazolo[4,3-a] pyrimidinones. Turk J Chem 2015;39:510–31.

[49] Frederick CA, Williams LD, Ughetto G, et al. Structural comparison of anticancer drug-DNA complexes: adriamycin and daunomycin. Biochemistry 1990;29:2538–49. 2334681

