# Supplementary material for: Design, synthesis, molecular modelling, and biological evaluation of novel substituted pyrimidine derivatives as potential anticancer agents for hepatocellular carcinoma
Source: J Enzyme Inhib Med Chem. 2019 May 23;34(1):1110–20. doi: 10.1080/14756366.2019.1612889 (PMC6537702; doi:10.1080/14756366.2019.1612889)
Supplement: Supplemental Material [file IENZ_A_1612889_SM8431.pdf]

Supplementary

Cell culture and treatment

All reagents were handled in a sterile fume hood. DMEM medium, and fetal bovine serum (FBS) were purchased from Gibco;phosphate-buffered saline pH 7.4 (PBS) and trypsin-EDTA were obtained from Sigmae Aldrich. Alamar Blue or Resazurin (Promega,Mannheim, Germany) reduction assay was used to assess the cytotoxicity of the studied samples. The growth medium (DMEMmedium with 10% FBS, 10 0 U/mL penicillin, and 10 0 mg/L streptomycin), and alamar blue were stored at 48 C, while trypsineEDTA and FBS were stored frozen at 208C and thawed before use; PBS was stored at room temperature. The Hep-G2, Huh-7 and normal fibroblast cells were obtained from the German Cancer Research Center (DKFZ).Cells were cultured in 50 cm2 culture flask s (Corning) using DMEM medium supplemented with 10% FBS, penicillin (10 0 IU/mL), and streptomycin (10 0 mg/mL). The culture was maintained at 37 C atmosphere of 5% CO2 and 95% relative humidity. The cells were transferred to a new fl ask every 2 days and treated with trypsineEDTA to detach them from the flask . Cells were counted under a microscope using a hemacytometer (Hausser Scientific).Cell solutions were diluted with growth medium to a concentration of 1 $*10^5$ cells/mL and transferred to a 96-well plate, and treated with gradient concentrations of test compounds.

Resazurin cell growth inhibition assay

Alamar Blue or Resazurin (Promega, Mannheim, Germany) reduction assay was used to assess the cytotoxicity of the studied samples. The assay tests cellular viability and mitochondrial function. Briefly y, adherent cells were grown in tissue culture flask s, and then harvested by treating the fl asks with 0.025% trypsin and0.25 mM EDTA for 5 min. Once detached, cells were washed, counted and an aliquot (5 * 10^3cells) was placed in each well of a96-well cell culture plate in a total volume of 100 mL. Cells were allowed to attach overnight and then treated with samples. The final concentration of samples ranged from 0 to 10 0 mM. After 4 8 h,20mL Resazurin 0.01% w/v solution was added to each well and the plates were incubated at 37C for 1 -2 h. Fluorescence was measured on an automated 96-well Infinite M2000 Pro^{TM} plate reader (Tecan, Crailsheim, Germany) using an excitation wave-length of 54 4 nm and an emission wavelength of 590 nm. Doxorubicin was used as positive control. Each assay was done at least three times, with two replicates each. The viability was compared based on a comparison with untreated cells. IC50(on cancer cells) were the concentration of sample required to inhibit 50% of the cell proliferation and were calculated from a calibration curve by a linear regression using Microsoft Excel

4.2.3. Caspase-glo 3/7 assay

The influence of our test samples on caspase 3/7 activity in pancreatic cancer resistant cells (Panc-1) was detected using Caspase-Glo 3/7 Assay kit (Promega). Cells cultured in DMEM were seeded in 96-well plates and treated with the sample (2 IC50;IC50;½ IC50) or DMSO (solvent control). After 24 h treatment,10 0 mL of caspase 3/7 reagent were added to each well, mixed and incubated for 1 h at room temperature. Luminescence was measured using well Infinite M2000 Pro^{TM} instrument (Tecan).Caspase 3/7 activity was expressed as percentage of the untreated control.
